# The Mechanism of Whey Protein Hydrolysates with Oral Immunotolerance

**DOI:** 10.3390/ijms27198637

**Published:** 2026-09-27

**Authors:** Meng-Yue Wang, Peng-Mei An, Lin Mao, Wen-Xuan Zhao, Yan-Jun Cong

**Affiliations:** Beijing Engineering and Technology Research Center of Food Additives, College of Food and Health, Beijing Technology and Business University, Beijing 100048, China; wmy20020621@163.com (M.-Y.W.); an18701249218@163.com (P.-M.A.); maolin1640@163.com (L.M.); 1863443697@163.com (W.-X.Z.)

**Keywords:** whey protein hydrolysate, oral immune tolerance, animal experiment, mechanism

## Abstract

To investigate the mechanism of oral immune tolerance induced by whey protein hydrolysates, this study utilized five industrially used proteases to hydrolyze whey protein and prepared the hydrolysates. An oral immune tolerance model was established using Balb/c mice. The levels of specific IgG, IgE, and histamine in the serum of mice from the five hydrolysate-preventive groups were measured. The expression levels of genes and proteins associated with the MAPK signaling pathway in small intestinal mast cells were analyzed using qPCR and Western blot. The results demonstrated that the expression of JNK in the jejunum from the trypsin, bromelain, papain, Neutrase, and pepsin mouse groups was downregulated, which correlated with the alleviation of allergic responses. A moderate increase in ERK expression was accompanied by an expansion of Treg cells, thereby inducing oral immune tolerance. The process of oral immune tolerance was regulated by the MAPK signaling pathway. This study systematically elucidates the oral immune tolerance mechanisms of the five hydrolysates, and the conclusions will provide the guidance for the development of hypoallergenic infant formula.

## 1. Introduction

According to statistics from the World Health Organization (WHO), food allergies have currently risen to sixth place among global diseases, with the number of people suffering from such conditions increasing exponentially, affecting over 20% of the global population, especially children, and becoming the most important non-communicable diseases affecting children’s health [1,2]. In 2023, the U.S. Food and Drug Administration (USFDA) updated and confirmed the nine major food allergens, which include cow’s milk, eggs, fish, shellfish, soy, tree nuts, peanuts, wheat, and sesame [3]. A series of survey results indicate that allergic reactions in early life are primarily due to cow’s milk and egg allergies [4,5,6,7,8,9,10].

Whey protein is a by-product of cheese production from cow’s milk and is a very important functional ingredient widely used in the food and medical industries, including infant formula, baked goods, sports nutrition, and medical foods. It mainly consists of β-lactoglobulin (50–55%), α-lactalbumin (20–25%), glycomacropeptide (10–15%), bovine serum albumin (5–10%), immunoglobulins (10–15%), lactoferrin (1–2%), and small amounts of trace components, rich in essential amino acids, branched-chain amino acids, and sulfur-containing amino acids [11]. Among these, β-lactoglobulin and α-lactalbumin are considered the major allergens [12,13].

Previous research by our team found that the trypsin hydrolysate of β-lactoglobulin in whey protein was rich in T-cell epitope peptides and possessed oral immunological tolerance characteristics. Based on these findings, this study prepared whey protein hydrolysates (WPH) and revealed the mechanism of oral immune tolerance through animal experiments.

## 2. Results

### 2.1. DH and Peptide Concentrations of Whey Protein Hydrolysates

#### 2.1.1. The DH of Whey Protein Hydrolysates

The DH of each hydrolysate was measured by the OPA method, and the results are shown in Figure 1. As shown in Figure 1, the hydrolysates obtained with pepsin and trypsin exhibited higher DH values, reaching 32.47% and 40.67%, respectively. Bromelain yielded a lower DH of 11.15%. The DH values for papain and Neutrase hydrolysates were 23.95% and 25.39%, respectively.

#### 2.1.2. The Peptide Concentrations of Whey Protein Hydrolysates

The peptide concentrations of the different enzymatic hydrolysates were determined using the Biuret method, and the results are presented in Figure 2. The peptide concentrations of the hydrolysates are as follows: pepsin hydrolysate, 0.739 mg/mL; papain hydrolysate, 0.657 mg/mL; bromelain hydrolysate, 0.546 mg/mL; neutral protease hydrolysate, 0.620 mg/mL; and trypsin hydrolysate 0.629 mg/mL.

### 2.2. Mass Spectrometry Analysis of Hydrolyzed Peptide Amino Acid Sequence Alignment Results

As shown in Table S1, six trypsin-hydrolyzed peptide segments partially or fully overlap with predicted β-lactoglobulin T-cell epitopes; 16 bromelain-hydrolyzed peptide segments partially or fully overlap with β-lactoglobulin epitopes, and one partially overlaps with α-lactalbumin epitopes; 16 papain-hydrolyzed peptide segments partially overlap with β-lactoglobulin epitopes, and one partially overlaps with α-lactalbumin epitopes; seven neutral protease-hydrolyzed peptide segments partially or fully overlap with β-lactoglobulin epitopes; and eight pepsin-hydrolyzed peptide segments partially or fully overlap with β-lactoglobulin epitopes, and one partially overlaps with α-lactalbumin epitopes.

### 2.3. Weight Changes and Clinical Symptom Scores of Mice in Each Experimental Group

#### 2.3.1. Weight Changes in Mice in Each Experimental Group

For mouse body weight data collected through multiple repeated measurements during the experiment, a two-way repeated-measures ANOVA was employed, with group as the between-group factor and time as the within-group factor. Figure 3 shows the body weight changes in mice during the experimental period. No significant differences in body weight were observed among the mouse groups. All mice exhibited a consistent increasing trend in body weight until the end of the trial, indicating that different WPHs did not exert a noticeable impact on animal body weight.

#### 2.3.2. Clinical Symptom Scores of Mice in Each Group

On day 42, mice from each group were challenged, and their clinical symptoms were scored as shown in Table 1. Compared with the NC group, the clinical symptom scores were significantly higher in mice from the PC group, bromelain hydrolysate, papain hydrolysate, trypsin hydrolysate, and Neutrase hydrolysate groups (*p* < 0.05), while there was no significant difference in the allergic symptom score in the pepsin hydrolysate group.

### 2.4. Level of Serum-Specific IgG

As shown in Figure 4, the levels of specific IgG in the serum of mice from the bromelain, pepsin, trypsin, Neutrase, and papain hydrolysate groups were significantly lower than those in the PC group (*p* < 0.05). These results indicate that WPHs produced by bromelain, pepsin, trypsin, Neutrase, and papain reduced sensitization responses in mice. Furthermore, continuous gavage of whey protein hydrolysate for 14 days induced a certain degree of oral tolerance in mice.

### 2.5. Level of Serum-Specific IgE

As shown in Figure 5, the IgE levels in the trypsin and pepsin hydrolysate mouse groups were significantly lower than those in the PC group (*p* < 0.05), while the IgE levels in the Neutrase, papain, and bromelain hydrolysate groups were significantly lower than those in the NC group (*p* < 0.05). These results indicate that WPHs produced by bromelain, pepsin, trypsin, Neutrase, and papain could induce a certain degree of oral tolerance in mice, with a more pronounced effect observed in the Neutrase, papain, and bromelain hydrolysate groups.

### 2.6. Level of Histamine in Serum

As shown in Figure 6, the histamine level in the serum of the PC mouse group was significantly higher than that of the NC mouse group. The histamine level in the bromelain hydrolysate group was between that of the PC and NC groups. This indicates that the inhibitory effect of bromelain hydrolysates on histamine release is relatively weak. While no significant differences were observed in the histamine levels of the trypsin, Neutrase, papain, and pepsin hydrolysate groups compared to the NC group. These four hydrolysis products can effectively inhibit histamine release in sensitized mice.

### 2.7. Histological Analysis of Mice Jejunum (H&E Staining)

The pathological images of the small intestine from mice treated with trypsin, bromelain, papain, neutral protease, and pepsin obtained by HE staining are shown in Figure 7.

According to the results of HE staining of the jejunum in various groups of mice, the PC group exhibited significant intestinal pathological damage, including an increased rate of shedding of intestinal villus epithelial cells and aggravated inflammatory cell infiltration, indicating that the undegraded whey protein caused an allergic reaction in the mice. There were no significant differences between the trypsin group and the NC group, with no lesions observed. The mice in the bromelain, papain, neutrophil elastase, and pepsin groups only exhibited a slight infiltration of neutrophils, with no obvious damage. This indicates that the WPHs administered through gavage for 14 consecutive days induced a certain level of oral immune tolerance in the mice, with trypsin and neutrophil elastase showing better effects.

### 2.8. Western Blot Analysis of the Expression Levels of p38MAPK, ERK, and JNK Proteins in Mice Jejunum

Western blot analysis was performed to explore the MAPK signaling pathway. As shown in Figure 8, the results indicated that after 14 consecutive days of oral administration of mice using WPH, the expression levels of p38MAPK in the jejunum from the trypsin, bromelain, papain, Neutrase, and pepsin mouse groups were significantly decreased (*p* < 0.05), suggesting that WPH could alleviate intestinal inflammation in mice and restore barrier function.

The levels of JNK in the jejunum were reduced in the trypsin, bromelain, papain, Neutrase, and pepsin mice groups, which may be associated with decreased mast cell degranulation and mitigated allergic responses. A moderate increase in ERK levels, potentially accompanied by an expansion of Treg cells (up-regulation of Foxp3 expression), may contribute to the induction of oral immune tolerance.

The grayscale analysis results demonstrated that all WPH (trypsin, bromelain, papain, neutrase, and pepsin) can induce a certain degree of oral immune tolerance in mice. Among these, trypsin and papain hydrolysates exhibited more pronounced effects.

### 2.9. qPCR Analysis of JNK and ERK Gene Expression Levels

The results of qPCR analysis revealed that the expression levels of JNK in the jejunum from the trypsin, bromelain, papain, Neutrase, and pepsin mice groups were significantly lower than those in the PC mice group (*p* < 0.05). The JNK expression levels in the five prevention mice groups were not significantly different from those in the NC group (Figure 9). In contrast, the expression levels of ERK in the jejunum of mice treated with trypsin, bromelain, papain, neutrase, and pepsin were significantly higher than those in the PC group (*p* < 0.01).

The activation of genes JNK and ERK genes in the intestinal tissue of mice may be related to the hydrolysates promoting T-cell proliferation and inhibiting allergy symptoms. This result preliminarily reveals the relationship between WPHs’ effects on intestinal cell function and the MAPK signaling pathway, providing crucial data support for the mechanism of WPH-induced oral immune tolerance.

## 3. Discussion

Gouw et al. reported that WPH containing T-cell epitopes enhanced immune tolerance in mice and effectively prevented bovine β-lactoglobulin allergy [14]. Yin et al. found that after gavaging allergic mice with WPH, the mice could restore Th1/Th2 cell balance, showing a significantly reduced allergic response; at the same time, WPH could promote the proliferation of beneficial gut microbiota in mice and increase the production of short-chain fatty acids (SCFAs), thereby enhancing intestinal immune tolerance and alleviating allergy symptoms [15]. On this basis, an oral immune tolerance model was established using WPHs in this study, and histopathological changes in the spleen and jejunum of the mice were analyzed, as well as the expression levels of genes and proteins related to the MAPK signaling pathway in mast cells, thereby providing an in-depth elucidation of the mechanism of oral immune tolerance.

Mass spectrometry results revealed a relatively high proportion of peptides in the trypsin hydrolysates that perfectly matched T-cell epitopes, whereas bromelain and papain generated more epitope-overlapping peptides, most of which were only partially overlapping. Additionally, the 40.67% DH of trypsin not only fully released the intrinsically hidden epitopes within the protein but also prevented excessive degradation of the epitope-containing peptides.

During the establishment of oral immune tolerance, specific IgG antibodies play a key regulatory role. They can prevent the activation of effector cells (such as mast cells and basophils) by inhibiting basophil activation, blocking the cross-linking of IgE with allergens, and binding to inhibitory receptors [16]. The results of this experiment showed that the IgG antibody levels in mice from the trypsin group, bromelain group, papain group, Neutrase group, and pepsin group were significantly increased. Moreover, the IgE antibody levels in mice from the bromelain group, papain group, and Neutrase group were significantly lower than those in the NC group. Histamine is an organic nitrogen-containing compound formed during the decarboxylation of histidine [17]. Histamine is abundant in mast cells of various tissues, especially in the skin, lungs, and intestinal mucosa. Histamine can be released due to tissue injury, inflammation, or allergic reactions. As an effective vasodilator, histamine can increase capillary permeability, leading to local tissue edema. Measuring the level of histamine release can reflect the intensity of an allergic reaction to some extent [18]. In this study, histamine release in mouse groups treated with trypsin, papain, neutral protease, or pepsin hydrolysates was significantly reduced, whereas the inhibitory effect of bromelain hydrolysates on histamine release was relatively limited. This may be attributed to the lower degree of hydrolysis by bromelain (DH = 11.15%, the lowest among the five enzymes) and the distinct composition of the peptides generated by this hydrolysis compared to those produced by the other four hydrolysates, thereby influencing its regulatory capacity over mast cell degranulation. Histopathological observations indicated that the jejunum of mice in the trypsin group showed no inflammatory response, and mice in the bromelain, papain, Neutrase, and pepsin treatment groups exhibited mild neutrophil infiltration in the jejunum.

The MAPK signaling pathway plays a central role in regulating immune responses, and its main members include JNK, p38 kinase, and ERK. p38 MAPK, as a key cellular mediator, is involved in the extracellular signal transduction induced by lipopolysaccharide, endotoxins, and pro-inflammatory cytokines (such as IL-1β and TNF-α) [19]. JNK and ERK affect allergic reactions and oral tolerance in the intestine through multiple mechanisms. The JNK signaling pathway regulates immune tolerance by modulating IL-10 production, while ERK suppresses allergic reactions by enhancing intestinal barrier function and promotes Treg cell differentiation and IL-10 secretion. Inhibiting JNK activity can enhance the induction of immune tolerance [20,21]. In this study, the expression levels of p38MAPK in the intestines of mice intervened with various protease-treated WPH were significantly reduced, indicating alleviation of intestinal inflammation and restoration of barrier function; the decrease in JNK levels was associated with reduced mast cell degranulation and alleviation of allergic symptoms; and the moderately elevated ERK levels may be related to an increase in Treg cells (upregulation of Foxp3 expression) [22]. Based on the findings of this study, this correlation suggests a potential link between ERK regulation and the induction of oral tolerance; however, this experiment has not yet provided direct evidence of changes in Treg/Foxp3 levels.

This study has the following limitations. First, due to experimental constraints, we did not directly measure Treg/Foxp3 expression or the degree of mast cell degranulation; the relevant immunomodulatory mechanisms were inferred indirectly based on the cytokine profile and symptom scores, and a direct causal relationship has not yet been established. Second, this study was conducted using a murine allergic model; however, mice and humans exhibit species-specific differences in their immune systems, gut microbiota, and metabolism. Therefore, the findings should be interpreted with caution when extrapolating them to the clinical application of hypoallergenic infant formula in humans; the potential clinical benefit requires further validation through subsequent human trials. Furthermore, the negative control group in this study received only cholera toxin (CT) without antigen sensitization. Future studies should include more stringent blank control groups.

## 4. Materials and Methods

### 4.1. Preparation of Whey Protein Hydrolysates

Whey protein (purity ≥ 90%; Shanghai Yuanye Biotechnology Co., Shanghai, China) was prepared as a 3% (*w*/*v*) aqueous solution. The optimal temperature and pH for the protease were determined (as shown in Table 2). The protease was added at a dosage of 1000 U/g protein, and the pH was adjusted to the appropriate value using 1 mol/L HCl or NaOH. The hydrolysis time was 2 h in a constant-temperature water bath shaker (Shanghai Longyue Instrument Equipment Co., Shanghai, China), during which the temperature and pH were maintained constant. After hydrolysis, the enzymatic reaction was immediately terminated by heating in a water bath at 85 °C for 10 min.

### 4.2. Determination of the Degree of Hydrolysis (DH) and Peptide Content

#### 4.2.1. Determination of the DH

The DH of the whey protein hydrolysate is was determined using the O-phthaldialdehyde (OPA) method [23]. The main principle is that when thiol groups are present, amino acids can undergo a specific reaction with OPA. The OPA reagent was prepared as follows: mix 10 mL of 50 mM OPA methanol solution, 10 mL of 50 mM N-acetylcysteine distilled water solution, 5 mL of 20% (*w*/*v*) SDS solution, and 75 mL of 0.1 M borate buffer solution with a pH of 9.5 were mixed thoroughly. The prepared OPA reagent should be stored in a light-proof manner, and it must be continuously stirred under light-proof conditions for 60 min before using.

During the sample testing, 3.2 mL of the freshly prepared OPA reagent was pipetted into a test tube, followed by the addition of 400 µL of the hydrolysate or L-leucine standard. The mixture was stirred uniformly and incubated at room temperature for 10 min. The absorbance of the hydrolysate or L-leucine standard was then measured at 340 nm using an ultraviolet spectrophotometer (Hitachi, Tokyo, Japan). A standard curve was constructed using L-leucine.

The total free amino group content of the whey protein was determined using an amino acid analyzer (Hitachi, Ltd., Japan) after hydrochloric acid hydrolysis.

DH was calculated using the following formula:

$$DH=\frac{(NH_{2})T_{x}-(NH_{2})T_{o}}{(NH_{2})T_{total}-(NH_{2})T_{o}}\times 100\%$$

where (*NH*_2_)*T_x_* is the free amino group content of the enzymatic hydrolysate (μmol/mL), (*NH*_2_) *T_o_* is the free amino group content of the unhydrolyzed whey protein (μmol/mL), and (*NH*_2_)*T_total_* is the total free amino group content in the whey protein sample (μmol/mL).

#### 4.2.2. Determination of Peptide Content

The peptide content of each hydrolysate was determined using the Biuret method [24].

### 4.3. Hydrolysis Mass Spectrometry Analysis Conditions

Weigh 1 mg of the sample precisely and prepare a protein solution at a concentration of 1 mg/mL; filter the solution through a 10 kDa membrane, concentrate it under vacuum to dryness, and then reconstitute it with a 0.1% formic acid aqueous solution. Adjust the concentration of all samples to 500 ng/μL; the sample loading volume is 2 μL. Label-free proteomics analysis and data interpretation for biological samples were performed using a nanoLC–Thermo QE Plus liquid chromatography–tandem high-resolution mass spectrometry system (Thermo Fisher Scientific, Waltham, MA, USA) coupled with the Proteome Discoverer 2.5 data processing software. LC-MS was employed to acquire MS1 and MS2 mass spectrometry data from the biological samples; protein identification was achieved by aligning the acquired peptide sequences against the Uniport protein database and performing peptide matching. Peptide separation was carried out using the Vanquish Neo nanoLC system; the analytical column was an Acclaim PepMap RSLC column (75 μm × 25 cm) (Thermo Fisher Scientific, Waltham, MA, USA), and the trapping column was an Acclaim PepMap 100 column (75 μm × 2 cm) (Thermo Fisher Scientific, Waltham, MA, USA). The mobile phase consisted of deionized water (Phase A) and an 80% acetonitrile–water solution (Phase B); the flow rate was 0.3 μL/min, and the sample injection volume was 1000 ng (2 μL). Mass spectrometry analysis was performed using a QE Plus high-resolution mass spectrometer (Thermo Fisher Scientific, Waltham, MA, USA) in Full-MS/DD-MS2 DDA mode, with a scanning range of 250–1800 *m*/*z*. The MS1 resolution was set to 70,000 and the AGC target value was set to 3 × 10^6^; the top 20 ions were selected for MS2 acquisition, with an MS2 resolution of 17,500 and an AGC target value of 1 × 10^5^; the NCE was set to 28, and the dynamic exclusion time was set to 25 s. The proteolytic sites used in mass spectrometry analysis are listed in Table 3.

### 4.4. Animal Experiment

The experimental procedure was adjusted with reference to [25]. Female BALB/c mice (3~4 weeks old) were purchased from Beijing Weitonglihua Company (“Beijing, China, permit number SCXK (Jing) 2021-0006”) were adaptively fed for 4 days in the SPF standard animal room. During the experiment, the mice were provided with free access to food and water, with the supplied diet confirmed to be free of allergens. The housing environment was strictly maintained at a temperature of 23 ± 3 °C and relative humidity of 40–70%, and a 12 h light/dark cycle. All mice used in this study were cared for in accordance with the Guidance for the Care and Use of Laboratory Animals published by the Ministry of Science and Technology of China (NCH Publication No. 11, revised in 2016), and all experimental studies were conducted under the program approved by the animal ethics committee of Beijing Laboratory Animal Research Center (License No.: BLARC-LAWER-202412002).

A total of 56 mice were randomly divided into seven experimental groups, with eight mice per group. The negative control (NC) mouse group received intragastric administration of saline, while the positive control (PC) mouse group received intragastric administration of whey protein. During days−14 to day 0, both the NC and PC groups were allowed free access to food and water. On days 0, 7, 14, 21, 28, and 35, the NC group received intragastric administration of normal saline containing 15 μg of cholera toxin (CT, Sigma-Aldrich, St. Louis, MO, USA), and the PC group received intragastric administration of 10 mg whey protein and 15 μg of CT (dissolved in normal saline). On day 42, the PC group was stimulated with 10 times the dose of whey protein without CT, and the NC group received intragastric administration of normal saline without CT.

During days −14 to 0, the five hydrolysates mice groups daily received intragastric administration of five different whey protein hydrolysates at a dose of 10 mg (dissolved in normal saline). On days 0, 7, 14, 21, 28, and 35, these groups received intragastric administration od 10 of mg whey protein and 15 μg CT (dissolved in normal saline). On day 42, after overnight fasting, the mice were given a high-dose challenge of 10 times the dose of whey protein (without CT). On day 21, blood was collected from the inner canthus vein in the eyes of the mice in each group to measure the titer of specific IgE antibodies and evaluate the degree of sensitization (Figure 10).

During the experiment, the growth conditions of the mice were monitored weekly, including their physical appearance, feeding behavior, and mental state. Body weight was accurately measured prior to each intragastric administration. Following the high-dose challenge, the condition of each mouse group was closely observed, and allergic symptoms occurring within 45 min after the challenge were recorded in detail and scored according to the following criteria: 0 points for no abnormal allergic symptoms; 1 point for mice showing nose-scratching or head-scratching behavior; 2 points for swelling around the eyes or mouth in mice, with bristled fur, along with reduced activity or increased breathing rate; 3 points for mice exhibiting asthma symptoms and experiencing difficulty in breathing; 4 points for mice having seizures or being in a state of immobility; 5 points for mouse death.

On the 42nd day, after high-dose stimulation and observation for 45 min, sample collection was performed immediately. Blood was collected from the inner canthus vein of the eyes of mice in each group, centrifuged to prepare serum, and antibody and histamine levels were measured. A segment of jejunal tissue was taken from 8 to 10 cm below the stomach to prepare jejunal pathological sections for analyzing the extent of lesions (×200). The mitogen-activated protein kinase (MAPK) cell signaling pathway in intestinal mast cells was also determined.

### 4.5. Determination of Serum-Specific IgG

The determination of specific IgG antibodies in mouse serum was performed using an indirect enzyme-linked immunosorbent assay and improved based on the literature [26]. In short, using 10 μg/mL of whey protein as the antigen, mouse serum diluted to 1:3200 as the primary antibody, and HRP-goat anti-mouse IgG diluted to 1:1000 as the secondary antibody, the optical density (OD) value was measured at a wavelength of 450 nm.

### 4.6. Determination of Serum-Specific IgE

Serum-specific IgE in mice was detected using an ELISA kit (Solabao Technology Co., Beijing, China) according to the manufacturer’s instructions.

### 4.7. Determination of Histamine in Mice Sera

The determination of histamine levels in mouse sera was performed using a histamine ELISA kit (Beijing Solabao Technology Co., Ltd., Beijing, China), following the manufacturer’s instructions.

### 4.8. Histological Analysis in Mice Jejunum

The experimental method was adjusted with reference to [27]. Hematoxylin-eosin (HE) staining was employed to observe the morphological structure and cellular infiltration of jejunal tissues.

### 4.9. Detection of p38MAPK, Extracellular Signal-Regulated Kinase (ERK), and c-Jun n-Terminal Kinase (JNK) Protein Expression Levels Using Western Blot

The experimental procedure was adjusted with reference to [28]. In short, take 10 mg of mouse intestinal tissue, grind it in liquid nitrogen, transfer it to a 1.5 mL centrifuge tube, add 150 μL of RIPA lysis buffer (containing protease inhibitors), sonicate for 30 s, centrifuge at 4 °C for 15 min, collect the supernatant, and determine the protein concentration using the Bradford assay.

First, perform SDS-PAGE electrophoresis with a stacking gel of 4% and a separating gel of 10%. Load 30 μL of the sample in each lane. Start with electrophoresis at 90 V for 30 min, then switch to 120 V for 2 h. Transfer at 300 mA for 2 h. Block with a protein-free rapid blocking solution for 5 min; then add the primary antibody (Mouse serum for all groups 1:1000) and incubate at 37°C for 1 h. Wash three times with TBST, 10 min each time. Incubate with the secondary antibody (HRP-labeled goat anti-mouse IgG 1:2000) for 1 h, wash two times with TBST, and finally rinse with TBS once for 10 min. Then lay the membrane flat, add ECL detection solution, and expose in a dark room for detection.

Evaluate the quantitative changes in band intensity using ImageJ1.54 software (NIH, Bethesda, MD, USA). Glyceraldehyde-3-phosphate dehydrogenase (GAPDH) was used as a loading control.

### 4.10. qPCR of JNK or ERK in Mice Jejunum

The experimental procedure was adjusted with reference to [29]. Total RNA was extracted from the mice jejunal tissue using the Trizol reagent kit (Bioss, Beijing, China). First-strand cDNA was synthesized from 2 μg of RNA using the TransScript^®^ One-Step gDNA Removal and cDNA Synthesis SuperMix (TransGen Biotech, Beijing, China). The PCR conditions were set for a total reaction volume of 15 μL, with an initial denaturation step at 95 °C lasting 10 min, followed by 40 cycles of amplification and quantification. The sequences of the primers for JNK, ERK and the GAPDH internal control are listed in Table 4. The relative gene expression levels were calculated using the 2-delta delta Ct (2^−△△Ct^) method.

### 4.11. Statistical Analysis of Data

All experimental data were derived from the mean values obtained from three or more replicate experiments. Graphs and correlation analyses were plotted using GraphPad Prism 9 software; significance testing was performed using one-way ANOVA and the Duncan post hoc test within SPSS 27.0 software; data marked with the same letter indicate no significant difference (*p* > 0.05). Prior to conducting ANOVA, the Shapiro–Wilk test was used to assess data normality, and Levene’s test was employed to check for homogeneity of variance. All data met the prerequisite assumptions required for parametric statistical analyses.

## 5. Conclusions

The results of this study demonstrate that certain proteolytic hydrolysates can effectively alleviate allergic symptoms induced by whey protein, as evidenced by reduced allergic manifestations, a significant decrease in IgE levels, and the absence of significant pathological damage. These findings provide supportive evidence for the induction of oral tolerance. However, these observed changes do not yet equate to the establishment of oral immune tolerance; the precise mechanisms underlying immune tolerance—such as the detection of Treg-specific markers and T-cell proliferation inhibition assays—require further investigation for confirmation. Therefore, the five whey protein hydrolysates can mitigate allergic reactions to whey protein in mice by regulating the expression of MAPK pathway-related proteins; their mode of action is consistent with the process involved in the induction of oral immune tolerance.

## Figures and Tables

**Figure 1:**
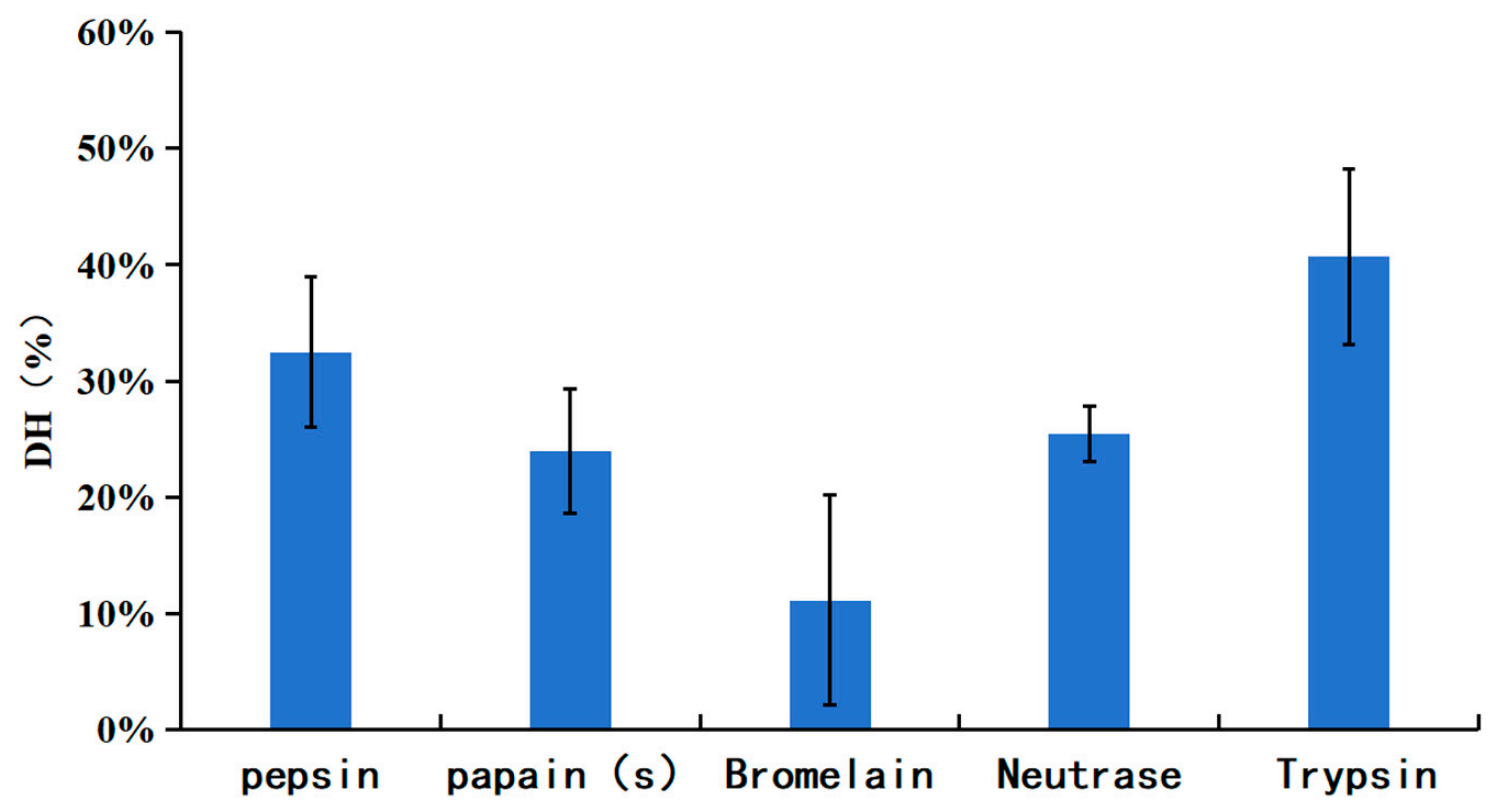
The DH of whey protein hydrolysates.

**Figure 2:**
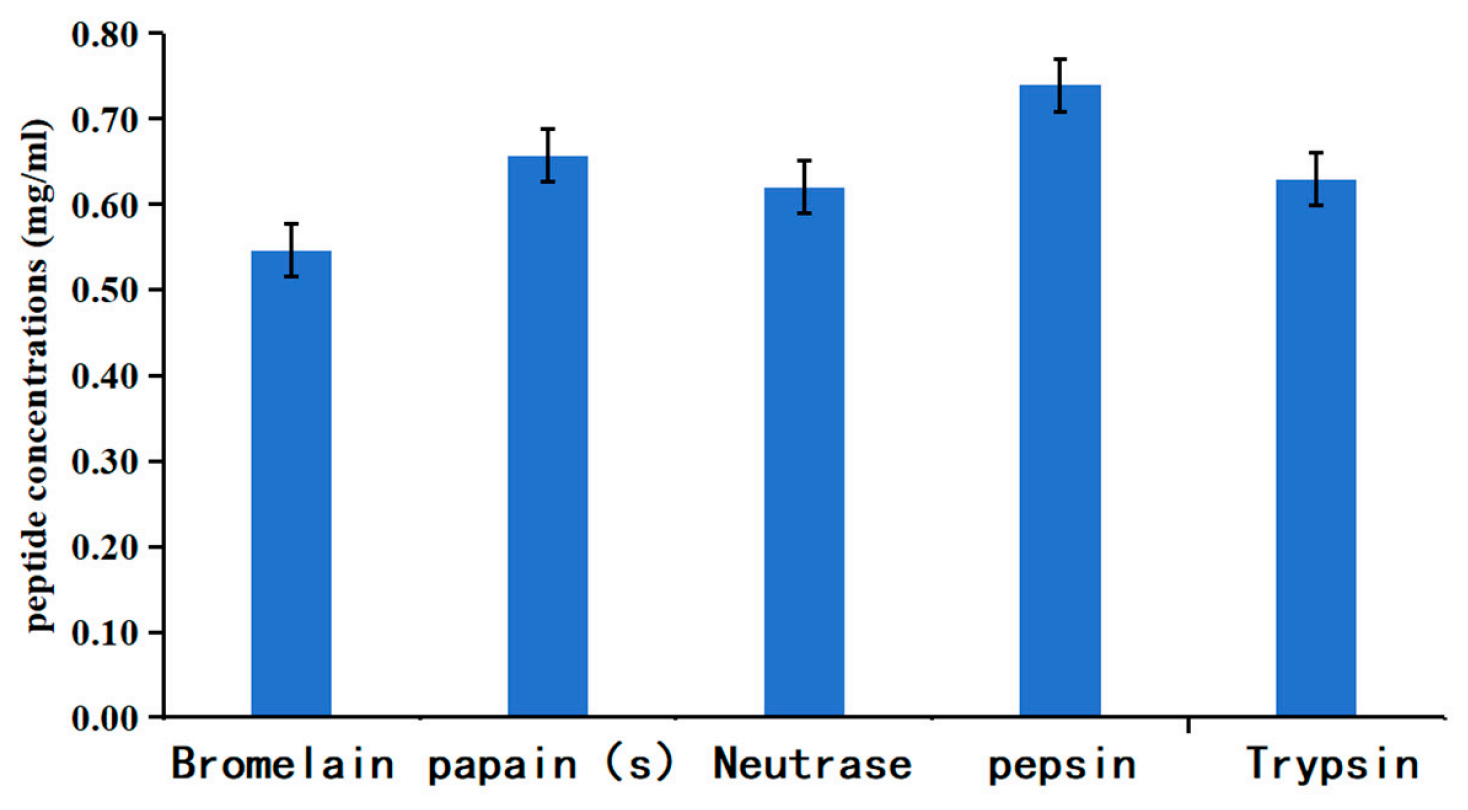
Peptide concentrations of whey protein hydrolysates.

**Figure 3:**
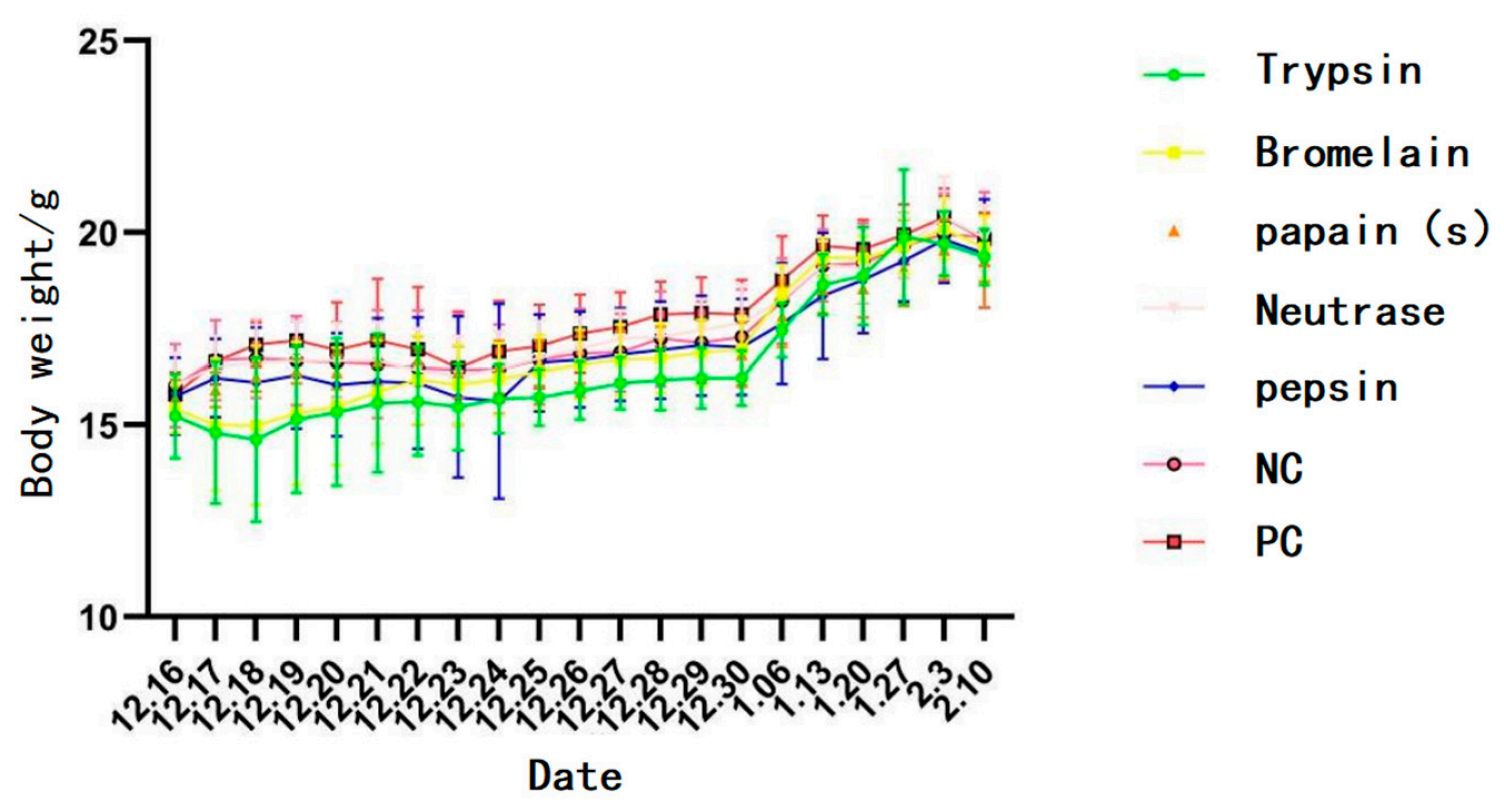
Weight changes in mice in each experimental group.

**Figure 4:**
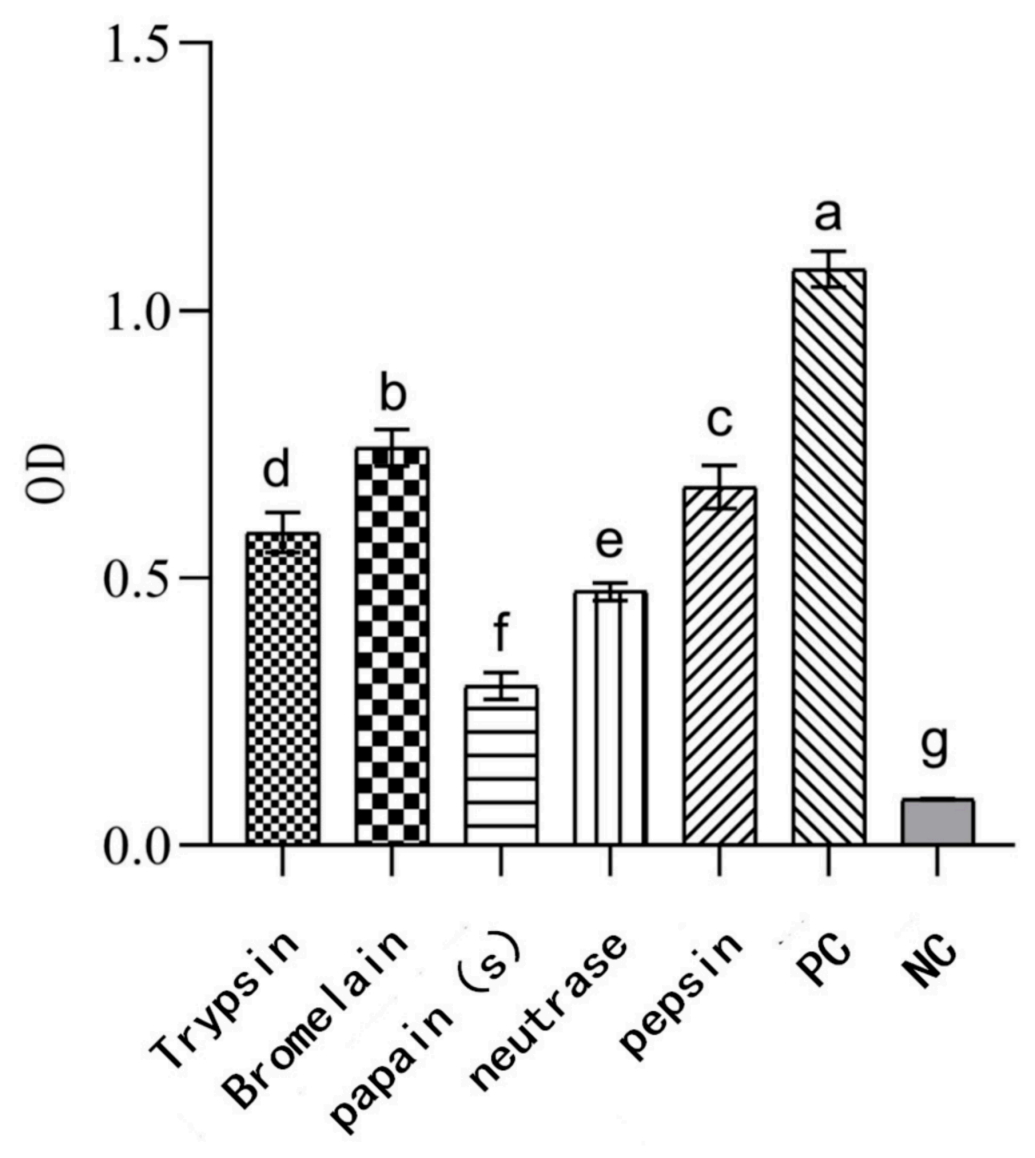
Specific IgG levels in the serum of mice from different groups. *n* = 8; Different letters indicate significant differences between groups.

**Figure 5:**
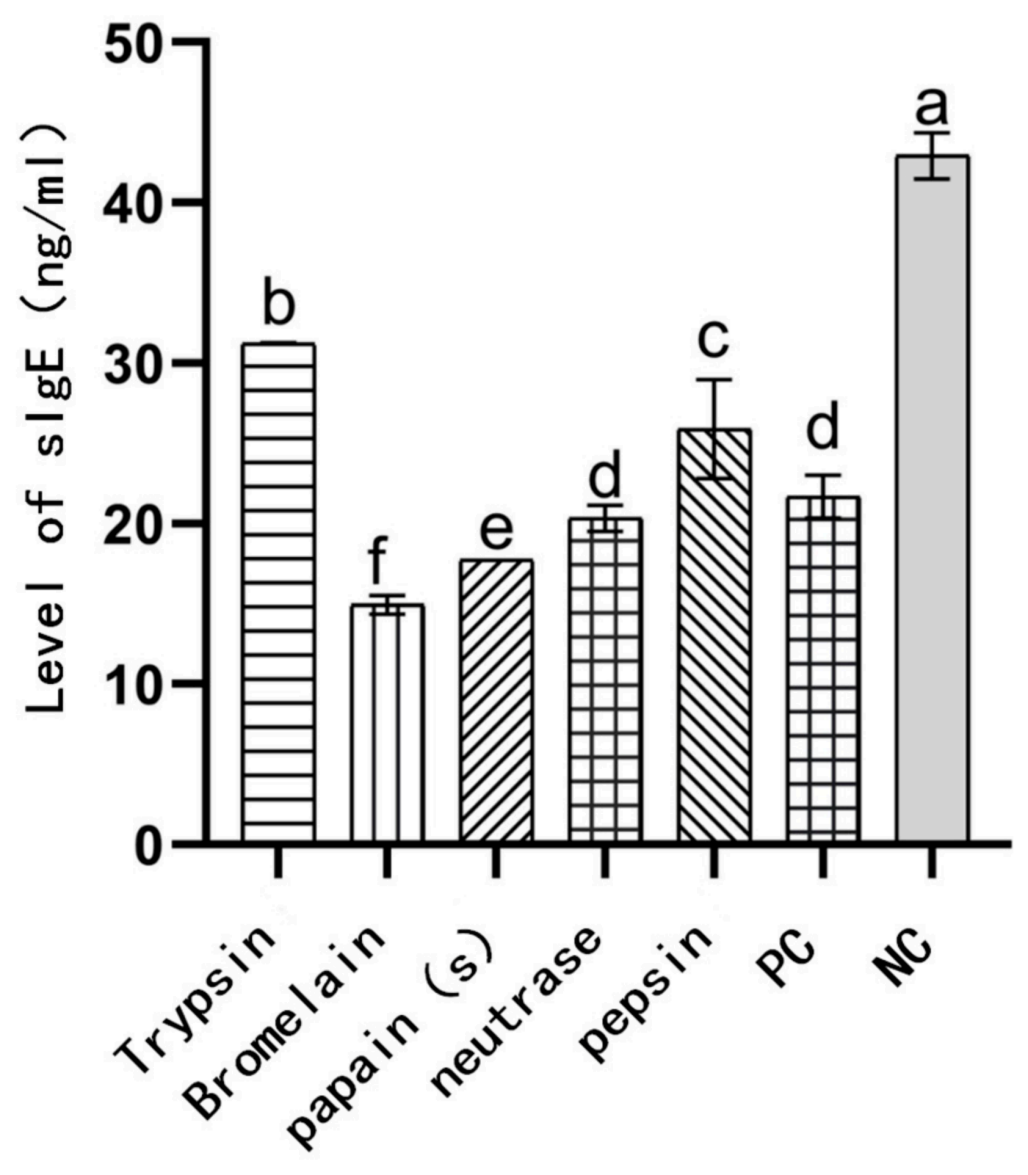
Specific IgE levels in the serum of mice from different groups. *n* = 8; Different letters indicate significant differences between groups.

**Figure 6:**
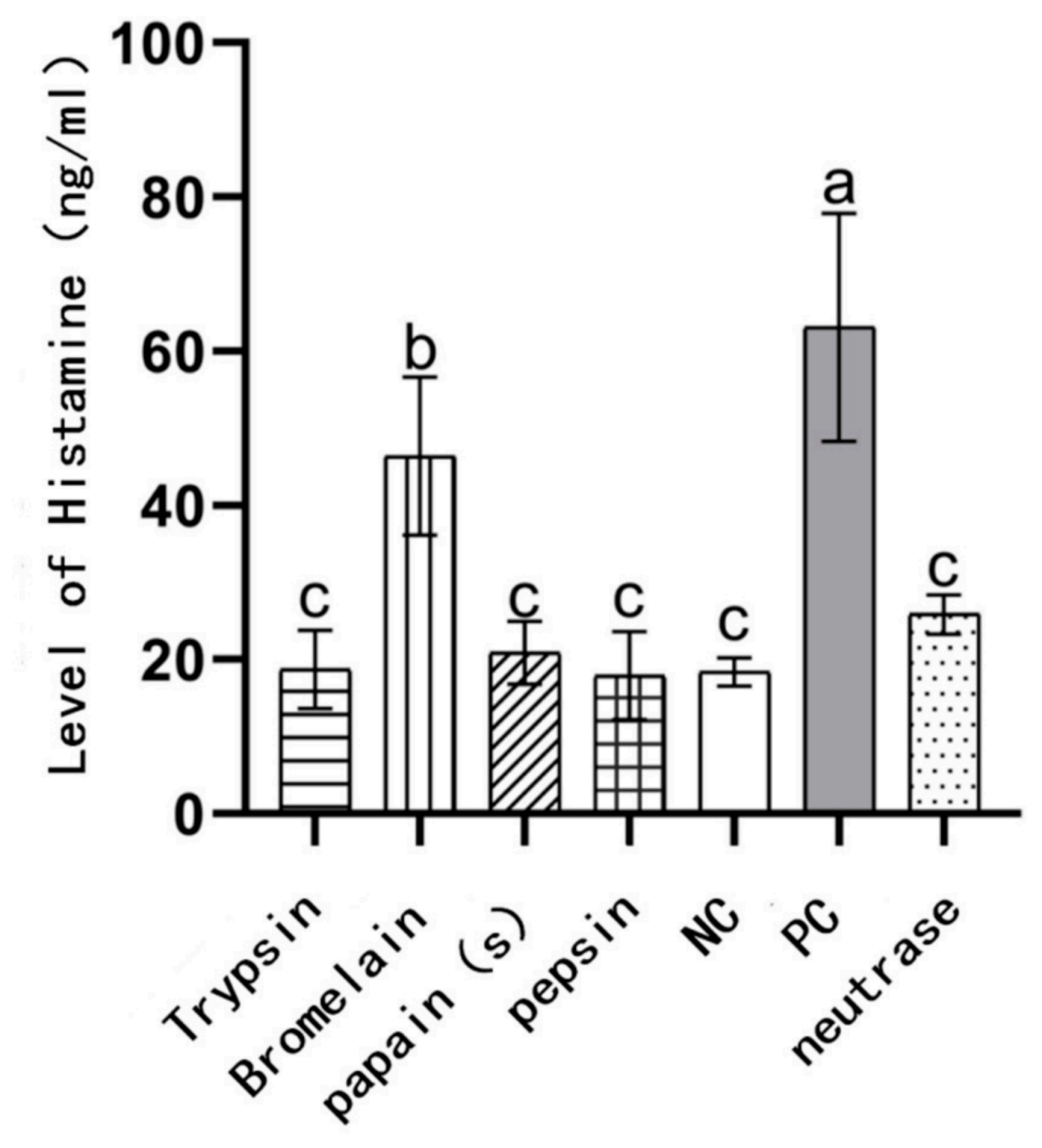
Histamine levels in the serum of mice from different groups. *n* = 8; Different letters indicate significant differences between groups.

**Figure 7:**
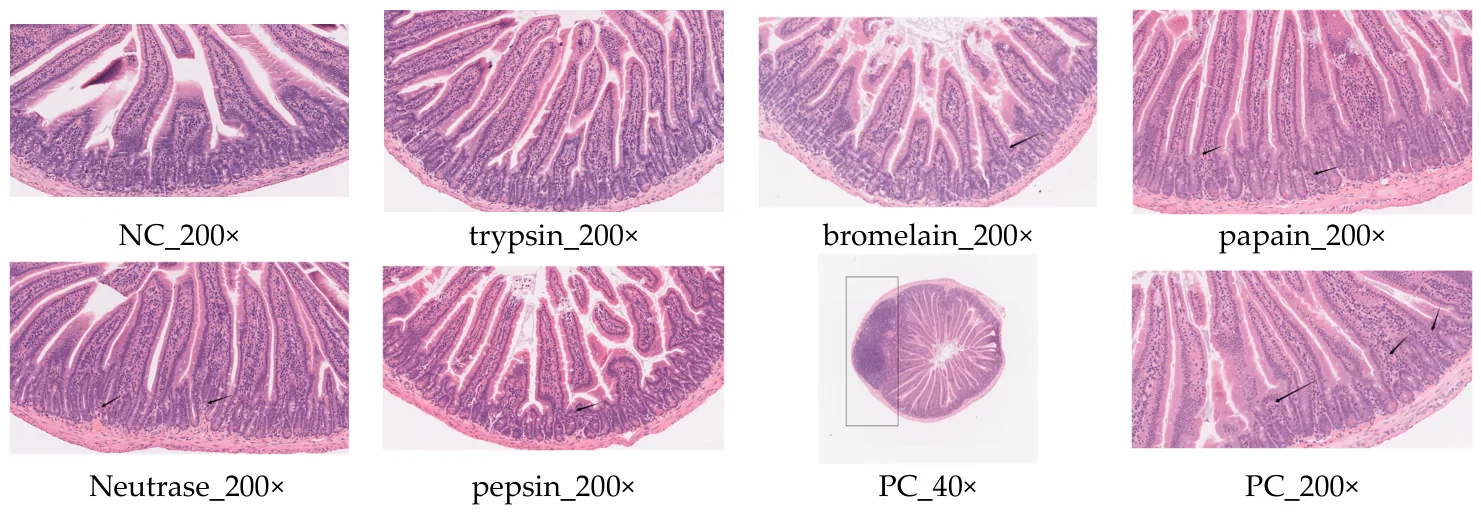
HE staining analysis of mice jejunum in each group.

**Figure 8:**
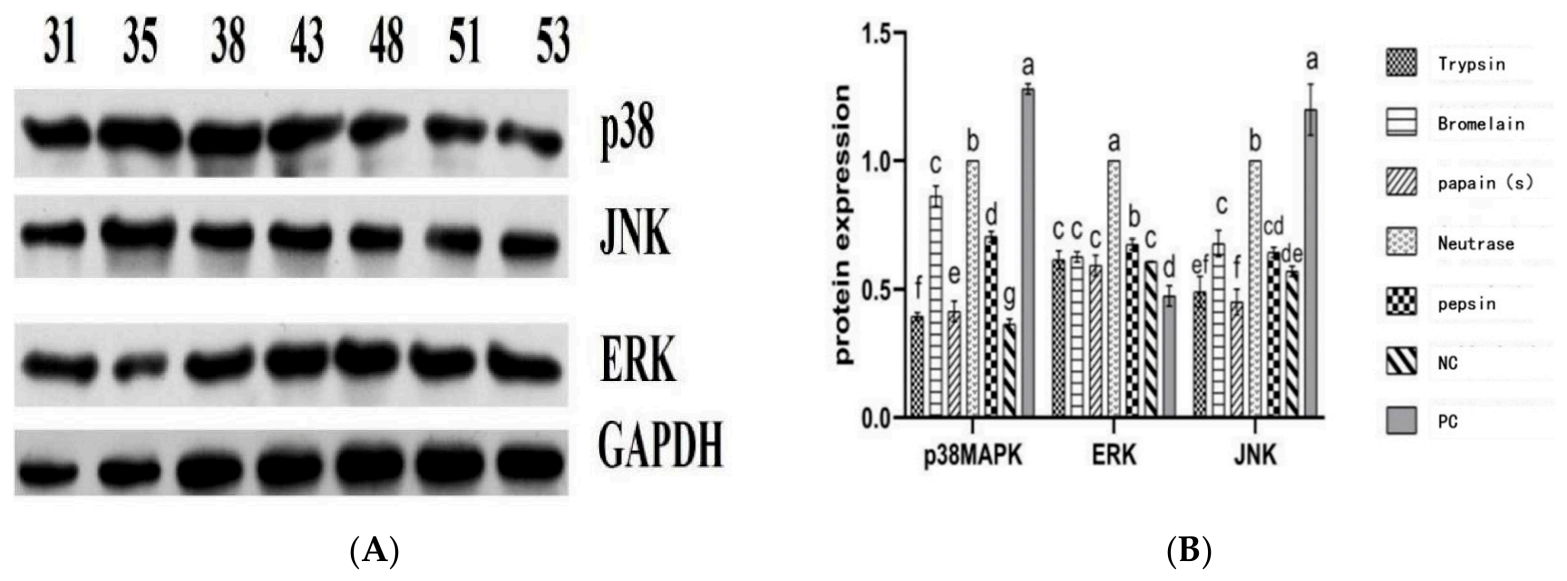
The expression levels of the MAPK pathway (ERK, JNK, p38MAPK) in the jejunal tissue of mice. *n* = 8; Different letters indicate significant differences between groups. (**A**) The expression levels of p38MAPK, JNK, and ERK in jejunal tissues were detected by Western blotting using GAPDH as the loading control; (**B**) quantification of p38MAPK, JNK, and ERK protein levels.

**Figure 9:**
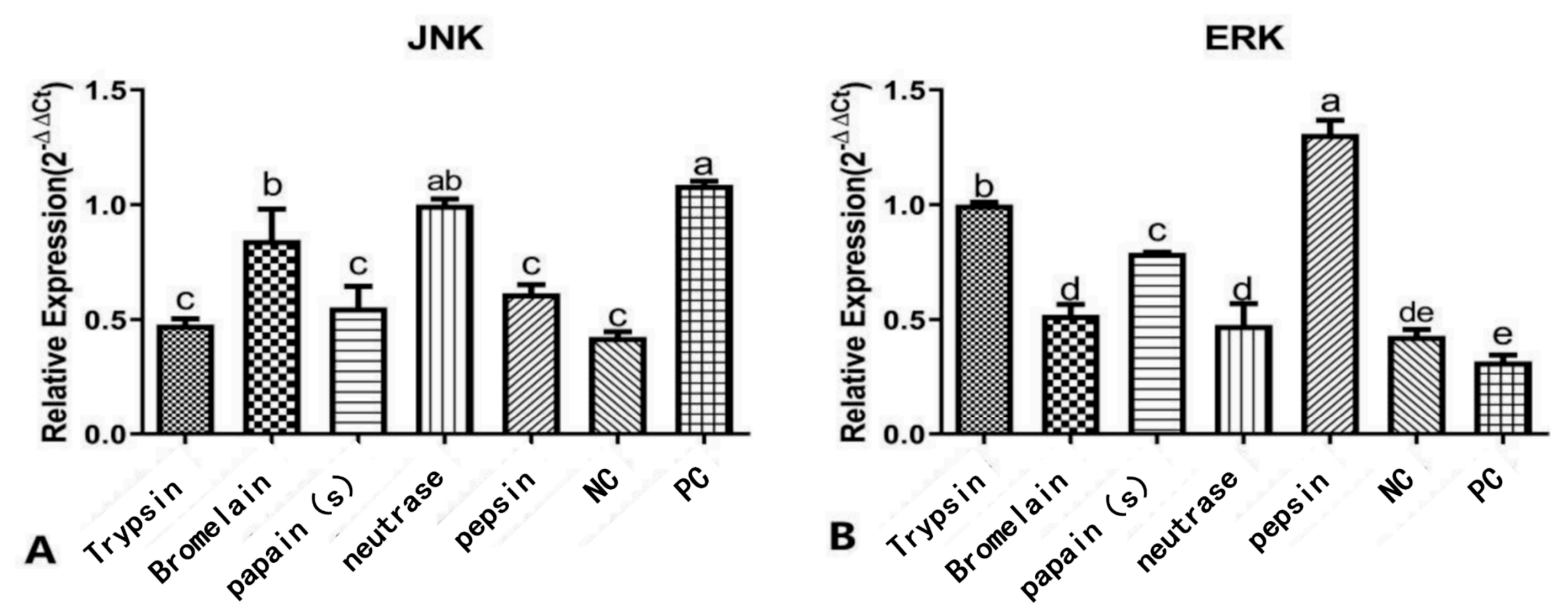
The expression levels of JNK and ERK genes in jejunal tissue of mice. *n* = 8; Different letters indicate significant differences between groups. (**A**) Quantitative Real-Time PCR Analysis of JNK Transcriptional Levels in Jejunal Tissue; (**B**) Quantitative Real-Time PCR Analysis of ERK Transcriptional Levels in Jejunal Tissue.

**Figure 10:**
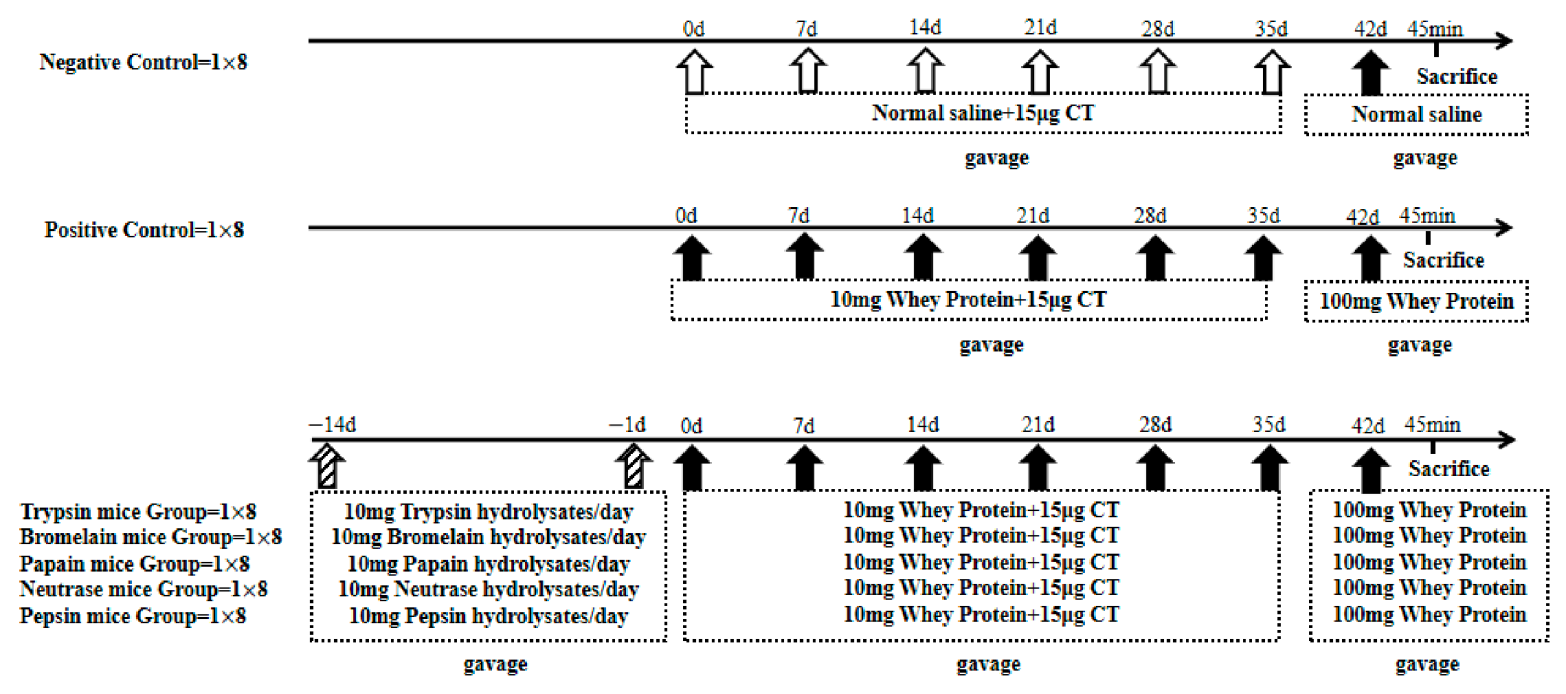
Schematic diagram of animal experiment.

**Table 1:** Clinical symptom scores of mice in each group. The results are expressed as the mean ± standard deviation; *n* = 8; Different letters indicate significant differences between groups.

Mice Groups	Scores of Clinical Symptoms
PC	2.750 ± 0.463 ^a^
Bromelain hydrolysate	1.625 ± 0.518 ^b^
Papain(s) hydrolysate	1.500 ± 0.535 ^b^
Trypsin hydrolysate	1.429 ± 0.535 ^b^
neutrase hydrolysate	1.250 ± 0.463 ^b^
pepsin hydrolysate	0.429 ± 0.535 ^c^
NC	0.125 ± 0.354 ^c^

**Table 2:** Optimal conditions for the hydrolysis of whey protein by various proteases.

Proteases	Substrate Concentration (%)	pH	Temperature (°C)
Trypsin	3	8.0	50
Bromelain	3	7.0	50
Papain(S)	3	7.0	60
Neutrase	3	7.0	50
Pepsin	3	2.0	37

**Table 3:** Protease hydrolysis sites.

Types of Enzymes	Enzymatic Cleavage Site
Neutrase	W, F, V, L
Papain(S)	L, G, K, R
Pepsin	F, L
Bromelin	K, A, Y, G
Trypsin	P, R, L

**Table 4:** Sequences of primers (JNK, ERK) and reference GAPDH.

Primer ID	Sequence (5′–3′)	Gene Name	Size (bp)
Q2851	aacgaccccttcattgacct	GAPDH	134
Q2852	tggaagatggtgatgggctt		
Q7412	gattttggactggcgaggac	JNK(Mapk8)	136
Q7413	tgcacccaactgaccaaatg		
Q7414	cagtctctgccctcgaaaac	ERK1(mapk3)	96
Q7415	gaaggttaacatccggtcca		

## Data Availability

The original contributions presented in this study are included in the article. Further inquiries can be directed to the corresponding author.

## Supplementary Materials

The following supporting information can be downloaded at: https://www.mdpi.com/article/10.3390/ijms27198637/s1.

## Abbreviations

The following abbreviations are used in this manuscript:

WPH	whey protein hydrolysates
DH	degree of hydrolysis
OPA	O-phthaldialdehyde
NC	negative control
PC	positive control
CT	cholera toxin
OD	optical density
HE	Hematoxylin-eosin
MAPK	mitogen-activated protein kinase
GAPDH	Glyceraldehyde-3-phosphate dehydrogenase
JNK	c-Jun N-terminal kinase
ERK	extracellular signal-regulated kinase
SCFAS	short-chain fatty acids
