# Supplementary material for: The Mechanism of Whey Protein Hydrolysates with Oral Immunotolerance
Source: Int J Mol Sci. 2026 Sep 27;27(19):8637. doi: 10.3390/ijms27198637 (PMC13664907; doi:10.3390/ijms27198637)
Supplement: Supplementary file 1 [file ijms-27-08637-s001.zip › ijms-4521251-supplementary.pdf]

Table S1. Results of amino acid sequence alignment for hydrolyzed peptide fragments obtained by mass spectrometry analysis

Protease Name	Allergen Name	**Mass spectrometry sequencing (amino acid localization)**	Prediction of T-cell epitope sequences (amino acid localization)	Mass spectrometry-based identification of sequences that partially or fully overlap with predicted T-cell epitope sequences	Is it a potential T-cell epitope?	Is it a newly discovered T-cell epitope?
Trypsin	β-LG	**DAQSAPLR（49-56)**	AASDISLLDAQSAPLRVYVEEL KPTP (41-66)	DAQSAPLR（49-56)	yes	Partially overlaps with previously reported AA24-50
		**AMAASDISLL (39-48)**	AASDISLLDAQSAPLRVYVEEL KPTP (41-66)	ASDISLL（41-48)	yes	Partially overlaps with the previously reported AA23-39 sequence
		**LSFNPTQL（165-172)**	DEALEKFDKALKALPMHIRLSF NPTQLEE（146-174)	LSFNPTQL (165-172)	yes	Partially overlaps with previously reported AA154-174
		**DAQSAPL（49-55)**	AASDISLLDAQSAPLRVYVEEL KPTP (41-66)	DAQSAPL（49-55)	yes	Partially overlaps with previously reported AA24-50
		**AMAASDISL（39-47)**	AASDISLLDAQSAPLRVYVEEL KPTP (41-66)	AASDISL（41-47)	yes	Partially overlaps with the previously reported AA23-39 sequence
		**SFNPTQL（166-172)**	DEALEKFDKALKALPMHIRLSF NPTQLEE（146-174)	SFNPTQL（166-172)	yes	Partially overlaps

						with previously reported AA154-174
Bromelin	β-LG	**ASDISLLDA（42-50）**	AASDISLLDAQSAPLRVYVEEL KPTP (41-66)	ASDISLLDA（42-50）	yes	Partially overlaps with the previously reported AA23-39 sequence
		**GLDIQK（25-30）**	QALIVTQTMKGLDIQKVA （15-32）	GLDIQK（25-30）	yes	Partially overlaps with previously reported AA1-20
		**GLDIQKVA（25-32）**	QALIVTQTMKGLDIQKVA （15-32）	GLDIQKVA（25-32）	yes	Partially overlaps with previously reported AA1-20
		**IDALNENK（100-107）**	ENKVLVLDTDYKKYL (105-119)	NK（105-107）	yes	Partially overlaps with the previously reported AA86-105
		**LDIQKVA（26-32）**	QALIVTQTMKGLDIQKVA （15-32）	LDIQKVA（26-32）	yes	Partially overlaps with previously reported AA11-30
		**LDIQKVAG（26-33）**	QALIVTQTMKGLDIQKVA （15-32）	LDIQKVA（26-32）	yes	Partially overlaps with previously reported AA11-30
		**LIVTQTMK（17-24）**	QALIVTQTMKGLDIQKVA （15-32）	LIVTQTMK（17-24）	yes	Partially overlaps with

						previously reported AA1-20
		**LIVTQTMKG（17-25）**	QALIVTQTMKGLDIQKVA（15-32）	LIVTQTMKG (17-25)	yes	Partially overlaps with previously reported AA1-20
		**IPAVFK（94-99）**	AQKKIIAEKTKIPAV（83-97）	IPAV（94-97）	yes	Partially overlaps with previously reported AA67–88
		**MAASDISLLDA（40-50）**	AASDISLLDAQSAPLRVYVEELKPTP (41-66)	AASDISLLDA（41-50）	yes	Partially overlaps with the previously reported AA23-39 sequence
		**SDISLLDA（43-50）**	AASDISLLDAQSAPLRVYVEELKPTP (41-66)	SDISLLDA（43-50）	yes	Partially overlaps with the previously reported AA34-52 sequence
		**SLAMAA（37-42）**	AASDISLLDAQSAPLRVYVEELKPTP (41-66)	AA（41-42）	yes	Partially overlaps with the previously reported AA29-45 sequence
		**TWYSLA（34-39）**	QALIVTQTMKGLDIQKVA（15-32）		no	
		**VAGTWY（31-36）**	QALIVTQTMKGLDIQKVA（15-32）	VA（31-32）	yes	Partially overlaps with previously reported

						AA11–20
		**VEELKPTPEGDLEILLQK（59-76）**	ASDISLLDAQSAPLRVYVEELKPTP (41-66)	VEELKPTP（59-66）	yes	Partially overlaps with previously reported AA42-56
		**VLVLDTDYK（108-116）**	ENKVLVLDTDYKKYL (105-119)	VLVLDTDYK（108-116）	yes	Partially overlaps with the previously reported AA110-128 sequence
		**VLVLDTDYKK（108-117）**	ENKVLVLDTDYKKYL (105-119)	VLVLDTDYKK（108-117）	yes	Partially overlaps with the previously reported AA110-128 sequence
	α-LA	**INYWLAHK（120-127）**	VKKILDKVGINYWLAHKALCSEK（111-133）	INYWLAHK（120-127）	yes	yes
Papain	β-LG	**AMAASDISL（39-47）**	AASDISLLDAQSAPLRVYVEELKPTP (41-66)	AASDISL（41-47）	yes	Partially overlaps with the previously reported AA23-39 sequence
		**AMAASDISLL（39-48）**	AASDISLLDAQSAPLRVYVEELKPTP (41-66)	AASDISLL（41-48）	yes	Partially overlaps with the previously reported AA23-39 sequence
		**DAQSAPL（49-55）**	AASDISLLDAQSAPLRVYVEELKPTP (41-66)	DAQSAPL（49-55）	yes	Partially overlaps with previously reported AA24-50
		**DAQSAPLR（49-56）**	AASDISLLDAQSAPLRVYVEEL	DAQSAPLR（49-56）	yes	Partially

			KPTP (41-66)			overlaps with previously reported AA24-50
		**DLEILL（69-74）**	AASDISLLDAQSAPLRVYVEEL KPTP (41-66)		no	
		**DIQKVAGTWYSL（27-38）**	QALIVTQTMKGLDIQKVA（15-32）	DIQKVA（27-32）	yes	Partially overlaps with previously reported AA11-30
		**DTDYKKYL (112-119)**	ENKVLVLDTDYKKYL (105-119)	DTDYKKYL（112-119）	yes	Partially overlaps with the previously reported AA110-128 sequence
		**IDALNENK（100-107）**	ENKVLVLDTDYKKYL (105-119)	ENK（105-107）	yes	Partially overlaps with the previously reported AA86-105
		**IDALNENKVL（100-109）**	ENKVLVLDTDYKKYL (105-119)	ENKVL（105-109）	yes	Partially overlaps with the previously reported AA86-105
		**LDAQSAPL（48-55）**	AASDISLLDAQSAPLRVYVEEL KPTP (41-66)	LDAQSAPL（48-55）	yes	Partially overlaps with the previously reported AA23-39 sequence
		**LDAQSAPLR（48-56）**	AASDISLLDAQSAPLRVYVEEL KPTP (41-66)	LDAQSAPLR (48-56)	yes	Partially overlaps with previously reported

						AA24-50
		**LSFNPTQL（165-172）**	DEALEKFDKALKALPMHIRLSF NPTQLEE（146-174）	LSFNPTQL (165-172)	yes	Partially overlaps with previously reported AA154-174
		**IVTQTMKG（18-25）**	QALIVTQTMKGLDIQKVA （15-32）	IVTQTMKG（18-25）	yes	Partial overlap with previously reported AA1-21 sequence
		**NENKVL（104-109）**	ENKVLVLDTDYKKYL (105-119)	ENKVL（105-109）	yes	Partially overlaps with the previously reported AA86-105
		**NENKVLVL（104-111）**	ENKVLVLDTDYKKYL (105-119)	ENKVLVL（105-111）	yes	Partially overlaps with the previously reported AA86-105
		**SFNPTQL（166-172）**	DEALEKFDKALKALPMHIRLSF NPTQLEE（146-174）	SFNPTQL（166-172）	yes	Partially overlaps with previously reported AA154-174
		**VAGTWYSL（31-38）**	QALIVTQTMKGLDIQKVA （15-32）	VA（31-32）	yes	Partially overlaps with previously reported AA26-46
	α-LA	**ILDKVG（114-119）**	VKKILDKVGINYWLAHKALCS EK（111-133）	ILDKVG（114-119）	yes	yes
Neutrase	β-LG	**VLDTDYKKYL （110-119）**	ENKVLVLDTDYKKYL (105-119)	VLDTDYKKYL （110-119）	yes	Partially overlaps with the previously

						reported AA110-128 sequence
		**LDTDYKKYL （111-119）**	ENKVLVLDTDYKKYL (105-119)	LDTDYKKYL （111-119）	yes	Partially overlaps with the previously reported AA110-128 sequence
		**DTDYKKYL (112-119)**	ENKVLVLDTDYKKYL (105-119)	DTDYKKYL （112-119）	yes	Partially overlaps with the previously reported AA110-128 sequence
		**KPTPEGDLEILL （63-74）**	AASDISLLDAQSAPLRVYVEEL KPTP (41-66)	KPTP （63-66）	yes	Partially overlaps with the previously reported AA47-67 sequence
		**DTDYKKYLLF （112-121）**	ENKVLVLDTDYKKYL (105-119)	DTDYKKYL （112-119）	yes	Partially overlaps with the previously reported AA86-105
		**NENKVLV（104-110）**	ENKVLVLDTDYKKYL (105-119)	ENKVLV （105-110）	yes	Partially overlaps with the previously reported AA86-105
		**NENKVL（104-109）**	ENKVLVLDTDYKKYL (105-119)	ENKVL （105-109）	yes	Partially overlaps with the previously reported AA86-105
Pepsin	β-LG	**LDTDYKKYLL （111-120）**	ENKVLVLDTDYKKYL (105-119)	LDTDYKKYL （111-119）	yes	Partially overlaps

						with the previously reported AA86-105
		**KPTPEGDL（63-70）**	AASDISLLDAQSAPLRVYVEEL KPTP (41-66)	KPTP（63-66）	yes	Partially overlaps with the previously reported AA47-67 sequence
		**DTDYKKYLLF（112-121）**	ENKVLVLDTDYKKYL（105-119）	DTDYKKYL（112-119）	yes	Partially overlaps with the previously reported AA86-105
		**SFNPTQL（166-172）**	DEALEKFDKALKALPMHIRLSF NPTQLEE（146-174）	SFNPTQL（166-172）	yes	Partially overlaps with previously reported AA154-174
		**DTDYKKYLL（112-120）**	ENKVLVLDTDYKKYL（105-119）	DTDYKKYL（112-119）	yes	Partially overlaps with the previously reported AA86-105
		**QKVAGTW（29-35）**	QALIVTQTMKGLDIQKVA（15-32）	QKVA（29-32）	yes	Partially overlaps with the previously reported AA29-45 sequence
		**DTDYKKYL（112-119）**	ENKVLVLDTDYKKYL（105-119）	DTDYKKYL（112-119）	yes	Partially overlaps with the previously reported AA86-105

		**IVTQTM（18-23）**	QALIVTQTMKGLDIQKVA（15-32）	IVTQTM（18-23）	yes	Partially overlaps with previously reported AA1-20
	α-LA	**IVQNNDSTEYGL（60-71）**	QAIVQNNDSTEYG（58-70）	IVQNNDSTEYG（60-70）	yes	Partially overlaps with the previously reported AA29-51 sequence

The gene and amino acid sequence information for α-lactalbumin was retrieved from the National Center for Biotechnology Information (NCBI) public database; the accession number is CAA29664.1, and the full-length amino acid sequence consists of 142 residues.

The gene and amino acid sequence information for β-lactoglobulin was obtained from the National Center for Biotechnology Information (NCBI) public database; the accession number is CAA32835.1, and the full-length amino acid sequence consists of 178 residues.

Retrieve amino acid sequences from NCBI and use the online tools IEDB (http://www.iedb.org/) or NetMHC II/pan4.0 (https://services.healthtech.dtu.dk/service.php?NetMHCIIpan-4.0) to predict T-cell epitopes.

On the IEDB homepage, first select the "T-cell epitope prediction" section and then choose the "MHC II Banding" option. Upload a Fasta file containing the α-lactalbumin sequence, select the HLA-DRB5 human gene as the pre-bound MHC molecule, and choose the NetMHCIIpan 4.1 EL (Recommended Epitope Predictor – 2023.09) as the prediction method. Set the peptide length to 13 amino acids; the potential α-LA T-cell epitopes predicted by this database can be retrieved using this online tool. In the final output results, a lower percentile rank value indicates a higher likelihood that the corresponding peptide represents a potential T-cell epitope. In this study, peptides with a Rank ≤ 12% are considered potential T-cell epitope peptides.

On the NetMHC II/pan4.0 web interface, enter the α-LA amino acid sequence, select the HLA-DRB5 human gene as the peptide candidate for binding to MHC molecules, and set the peptide length to 13 amino acids. In the final output, a higher score indicates a greater likelihood that the peptide represents a T-cell epitope. In this

study, any peptide with an output score ⩾ 0.2 was considered a potential T-cell epitope peptide.

Enter the β-lactoglobulin amino acid sequence into either of the two bioinformatics tools, or upload a Fasta file containing the β-lactoglobulin sequence, to predict the T-cell epitopes of β-lactoglobulin.

The T-cell epitope identification results for α-lactalbumin obtained from the IEDB database indicate that these epitopes are primarily concentrated within five amino acid sequence segments: AA9 – 24, AA25 – 45, AA58 – 70, AA66 – 79, and AA111 – 133. Using NetMHC II/pan4.0 for prediction, the T-cell epitopes of α-lactalbumin were identified as being concentrated within three amino acid sequence segments: AA10 – 25, AA25 – 44, and AA116 – 131. By combining the results from both prediction methods, it can be concluded that the T-cell epitope regions of bovine α-lactalbumin are located within the following five amino acid sequence segments: AA9 – 24, AA25 – 45, AA58 – 70, AA66 – 79, and AA111 – 133.

The T-cell epitope identification results for β-LG obtained from the IEDB database indicate that these T-cell epitopes are primarily concentrated within four amino acid sequence segments: AA15 – 32, AA41 – 66, AA83 – 119, and AA146 – 174. The T-cell epitope predictions for β-LG using NetMHC II/pan4.0 also reveal that the T-cell epitopes are localized to four amino acid sequence segments: AA42 – 66, AA84 – 97, AA105 – 119, and AA146 – 166. By integrating the results from both prediction tools, it can be concluded that the T-cell epitope regions of bovine β-lactoglobulin span five amino acid sequence segments: AA15 – 32, AA41 – 66, AA83 – 97, AA105 – 119, and AA146 – 174.
